# A Mediation Moderation Model between Self-Evaluative Emotions and Relapse Rate among Polysubstance Users: A Comparative Cross-Sectional Study

**DOI:** 10.3390/ijerph20043164

**Published:** 2023-02-10

**Authors:** Mujahid Iqbal, Yu Yan, Na Zhao, Sumaira Mubarik, Silu Shrestha, Muzzamel Hussain Imran, Samrah Jamshaid, Najam ul Hasan Abbasi

**Affiliations:** 1 Department of Psychology, School of Philosophy, Wuhan University, 185 Donghu Road, Wuhan 430072, China; 2 State Key Laboratory of Resources and Environmental Natural Resources Research, Chinese Academy of Sciences, Beijing 100101, China; 3 Department of Epidemiology and Biostatistics, School of Public Health, Wuhan University, 185 Donghu Road, Wuhan 430072, China; 4 Department of Religious Studies, School of Philosophy, Wuhan University, 185 Donghu Road, Wuhan 430072, China; 5 School of Psychology, Northeast Normal University, Changchun 130024, China; 6 Department of Education Sciences, Mianyang University, Mianyang 621000, China

**Keywords:** shame-proneness, guilt-proneness, relapse rate, self-efficacy, polysubstance users, single-substance users, comparative study

## Abstract

A substantial portion of drug abuse research has concentrated on people with a single-substance-use disorder (SSUD), but many people abuse more than one drug. Studies have yet to examine how those with polysubstance-use disorder (PSUD) differ from those with an SSUD on the risk of relapse, self-evaluative emotions (e.g., shame and guilt), and personality factors (e.g., self-efficacy). Eleven rehab facilities in Lahore city, Pakistan were randomly chosen to provide a sample of 402 males with PSUD. For comparison, 410 age-matched males with SSUD were enlisted using a demographic form with eight questions, the State Shame and Guilt Scale, and the General Self-Efficacy Scale. Mediated moderation analysis was performed using Hayes’ process macro. The results demonstrate that shame-proneness is positively associated with relapse rate. Guilt-proneness mediates the relationship between shame-proneness and relapse rate. Self-efficacy buffers the influence of shame-proneness on relapse rate. Although the mediation and moderation effects were found in both study groups, these effects were significantly stronger among people with PSUD than those with SSUD. To be more specific, people with PSUD reported a higher overall score on shame, guilt, and relapse rate. Additionally, people with SSUD indicated a higher score on self-efficacy than those with PSUD. The findings of this study suggest that drug rehab facilities should implement a variety of strategies to raise drug users’ levels of self-efficacy, which will help to reduce their risk of relapse.

## 1. Introduction

Polysubstance use has long been recognized as a significant clinical and public health problem, but in recent years, due to rising concerns, it has attracted special attention [1,2]. Although the majority of addiction research has concentrated on one particular substance of abuse, many persons abuse multiple substances [3]. There has not been much empirical study on people with polysubstance-use disorder (PSUD); however, some evidence suggests that PSUD may be increasing [4]. According to the limited research that has looked into the clinical effects of polysubstance use, there are links between higher rates of lifetime depression, deviant conduct, arrests, and medical, financial, and legal issues [5,6]. Multiple-substance use has been linked to riskier social behaviors, as well as more severe physical and psychological comorbidities [7]. People with PSUD are at a higher risk for medical and psychological disorders than those with single-substance-use disorder (SSUD) [1].

There are 7.6 million drug users in Pakistan, 78% of whom are men and the remaining 22% are women. The number of people with SUD is growing by 40,000 every year. Polysubstance use is common, with one in five reporting more than one controlled substance [8]. The study was restricted to male participants given the fact that SUD is significantly more prevalent in men where the study was conducted (i.e., 78% of those with SUD in Pakistan were male in 2013). Pakistan is one of the nations with the highest drug-abuse rates in the world. Therefore, it is essential to carry out an empirical investigation on males with SUD in Pakistan. Frequent use of numerous substances as a coping mechanism may favor the development and maintenance of PSUD [9]. Beyond cognitive aspects, self-evaluative emotions are a critical relapse component in substance-use disorder [10]. This is especially true for shame- and guilt-proneness, which are elicited by social norm transgressions. Shame is a negative evaluation that focuses on the self, while guilt focuses on the (problematic) behavior. For example, a shame-prone person may say “I am bad,” whereas a guilt-prone person may say “My behavior was bad.” Shame-proneness represents an individual’s overall susceptibility to feeling shame, and this is quite similar to guilt-proneness [11]. Alcohol abuse and substance-use problems are positively associated with shame-proneness and negatively with guilt-proneness. Moreover, lower guilt-proneness is related to higher drug use in adolescence [12,13]. Some other studies [14,15] examined shame and guilt-proneness in clinical populations, and they found that individuals with PSUD reported lower guilt-proneness and higher shame-proneness. Previous findings also indicate that higher shame-proneness is associated with relapse in people with SUD. In addition, shame has been identified as a major problem for alcoholics: it leads to relapse and resistance to treatment [16]. In a recent study, researchers in Pakistan examined the interaction between shame and guilt experienced by people who use multiple substances. The authors found that the PSUD population reported more frequent shame and guilt activations. The study also discovered a positive correlation between shame and guilt activations in polysubstance users [17]. These findings highlight the importance of self-evaluative emotions in substance-use disorders.

Although shame- and guilt-proneness are common in medical and psychiatric conditions [18,19], little research has been conducted on them in polysubstance users. In addition, a lack of evidence has been found on the comparison of relapse rate between people with PSUD and those with SSUD. These variables hardly ever appear alone; instead, they frequently do so in clusters that draw one another and serve as building blocks. It is yet unknown how shame propensity and the underlying systems interact. Since addiction is a very stressful disease, and stress plays a part in its effectiveness, no independent research has been done in Pakistan on self-evaluative emotions, self-efficacy, and relapse rate in people with PSUD.

**Figure 1:**
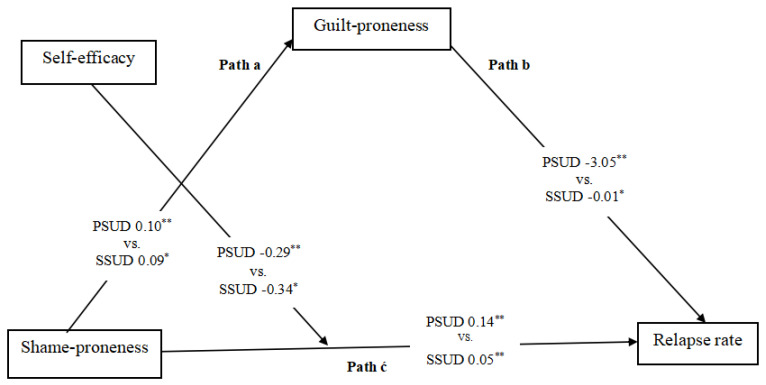
A conceptual framework and hypothesized model; * *p* < 0.05, ** *p* < 0.01.

On the other hand, the effects of potential moderators that link with relapse in substance-use disorder (SUD) are limited. Substance abuse is characterized by a persistent relapse stage. Although self-efficacy is an essential external component impacting relapse among people with PSUD, further research is required to determine how self-efficacy influences the occurrence of relapse behavior. The literature showed that self-efficacy is negatively associated with addiction relapse. A substance abuser with high self-efficacy is less likely to relapse after treatment [20,21]. The correlations between these factors can be determined using mediation and moderation methods, making it an appealing area for further research. Moreover, most research that has been conducted so far has been on people in Western countries who are going through voluntary detoxification and community drug treatment. So, research needs to be carried out on people with PSUD who are getting help in rehabilitation centers in Asia. In this study, we compare people with SUD who carried a diagnosis of PSUD to those diagnosed with SSUD, by considering: (a) whether shame-proneness directly predicts the relapse rate; (b) whether guilt-proneness plays a mediating role between shame-proneness and relapse rate; and (c) whether general self-efficacy plays a moderating role between shame-proneness and relapse rate. A research framework and hypothesized model is presented in Figure 1.

### 1.1. The Relationship between Shame-Proneness and Relapse Rate

Predisposition to feeling shame seems to increase the likelihood of developing substance-abuse problems. Since feelings of shame are often followed by avoidance behaviors such as isolation and substance abuse, it follows that shame may play a role in the onset of such issues [22]. According to the studies that have been conducted on people with PSUD, the predisposition for shame has been strongly associated with substance misuse [12,13,23]. Shame can both precede and cause substance misuse among people with SSUD (i.e., alcoholics). Further, shame is a significant barrier to recovery from alcoholism, and it also plays a role in people’s reluctance to seek help [24]. Previous research also shows that higher levels of shame are linked to relapse in people with PSUD [16,25].

Another study of people with SSUD contends that the tendency to relapse, the degree of that relapse, and impairments in mental and physical health are all predicted by shame about prior addictive behaviors [26]. Moreover, a study looked into how shame is related to alcohol use and associated issues. The authors concluded that those with higher shame-proneness consumed more alcohol and had more issues related to alcohol [27]. In addition, shame may act as a roadblock to recovery from SUD and it can be a risk factor for relapse [2]. In light of previous empirical findings, we proposed that:

**Hypothesis** **1.** *There would be a significant positive association between shame-proneness and relapse rate among people with PSUD.*

### 1.2. The Mediating Role of Guilt-Proneness

We proposed that shame positively predicts guilt, and guilt negatively predicts the risk of relapse. Previous studies on people with PSUD have shown that individuals who feel shame are more likely to feel guilty, and people who feel guilty are less likely to use drugs [10,23,28]. A study on people with SSUD showed that shame was positively associated with guilt, while guilt was positively correlated with protective behavior tactics after taking alcohol [29]. A longitudinal study on people with PSUD discovered that guilt-proneness was linked to less use of alcohol and other drugs in young adulthood [30]. In contrast, shame-proneness does not seem to provide the same level of protection against the development of SUD, and it may even encourage addictive behaviors [13]. According to a study on alcoholics, guilt-proneness is positively correlated with willingness to change [31].

Higher shame-proneness individuals consumed more alcohol and had more alcohol-related issues. On the contrary, guilt-prone people consumed less alcohol and had fewer issues related to alcohol [27]. The connection between a person’s shame and guilt propensity and their reaction to offenses was described by Tangney et al. (2009). The authors claimed that shame and guilt were positively associated with people with SUD. Further, someone who is highly guilt-prone could fail, feel embarrassed about it, and then take steps to make it right [32]. Findings thus imply that guilt is a type of negative affect that is adaptive. Guilt-proneness plays a mediating role in several other psychiatric conditions. For example, it plays a mediating role in the relationship between PTSD symptoms and anger among returning veterans [33], and guilt also mediates the impact of alexithymia on distress and suicide-related symptoms among males [34].

In short, individuals who are prone to shame frequently exhibit a variety of dysfunctional behaviors and physiologic results, such as depression, anxiety, eating disorders, persistent rage, increased cortisol reactivity, and compromised immune-system function [35]. However, the guilt-prone reported more anticipatory guilt and more anticipatory guilt associated with less engagement in heavy episodic drinking [36]. Recent research on polysubstance users in Pakistan claimed that shame positively predicted guilt [17]. Thus, in the present research, we expected to find that shame-proneness positively predicts guilt-proneness and guilt-prone individuals negatively predict relapse rate. Based on the above evidence, we expected the following:

**Hypothesis** **2.** *Guilt-proneness will mediate the relationship between shame-proneness and relapse rate among people with PSUD*.

#### The Moderating Role of Self-Efficacy

Self-efficacy theory, developed by [37], is an integrative paradigm that explains the psychological and behavioral alterations brought by various therapeutic modalities. Effective methods are thought to produce favorable results by raising individual efficacy expectations. These expectations are thought to affect how coping behavior is initiated, how much effort is put forth to maintain it, and how long it is maintained in the face of both internal and external challenges. The theory contends that the drive to achieve a specific result, contextual clues, and perceived self-efficacy all influence behavior.

Self-efficacy is the belief that one can take the necessary actions to achieve the desired result. For example, it alludes to the notion that one can stop using drugs and maintain abstinence. Self-efficacy has been receiving more attention as a mediator and/or moderator of treatment outcomes in a number of areas including addiction treatment [20]. Self-efficacy has been shown in many studies of treatment for drug abuse to be an important predictor of outcome or a way that treatment works. When they make a mistake, drug users with high self-efficacy tend to see it as a temporary setback and get back on track, while those with low self-efficacy are more likely to have a full-blown relapse [38]. So, this study was carried out to investigate how self-efficacy affects the relationship between shame-proneness and the rate of relapse.

Although there is a chronic relapsing cycle of substance use [39], prior research indicated that self-efficacy can prevent relapse in addiction [40,41]. Additionally, people with PSUD who have a high level of self-efficacy would have a reduced likelihood of relapsing into substance use [42,43]. Thus, a lack of self-efficacy among drug users is one of the most significant variables influencing the rate of relapse. According to Bandura, humans cannot be considered to be simply driven by internal urges or by external stimuli; rather, psychological functions control how people behave and react to their environment.

In order to successfully apply cognitive, social, emotional, and behavioral regulation to the accomplishment of diverse goals, Bandura contends that self-efficacy is a key component. People’s beliefs about their abilities to accomplish their tasks are effective, but knowledge, skills, and past achievements are not reliable predictors of future performance [37]. There are disparities in the combined power of talents, improper approaches, and conditions. People know what to do and have the necessary skills, but they do not always use them. Self-efficacy enables the transmission of information and talents through cognitive, motivational, and emotional processes. Self-efficacy is not a skill, but a conviction in the ability to work in varied settings. Self-efficacy theory suggests that treatment will be effective when the patient has acceptable expectations of what may be attained [44].

A review of the literature indicated that self-efficacy is consistently linked to stopping addictive behaviors and relapse. Therefore, based on self-efficacy theory and previous findings we proposed that:

**Hypothesis** **3.** *Self-efficacy will moderate the positive relationship between shame-proneness and relapse rate. Specifically, this relationship will be stronger for substance users who have lower (vs. higher) self-efficacy levels than for substance users with higher (vs. lower) self-efficacy levels.*

## 2. Material and Methods

### 2.1. Participants and Recruitment

A sample of *n* = 812 males with SUD was recruited from eleven rehabilitation centers in Lahore, Pakistan. For comparison purposes, we divided these participants into two groups. Of the 812, *n* = 402 were diagnosed with PSUD, with ages ranging from 18 to 65 years (*Mean* = 32, *SD* = 5.5), and *n* = 410 were diagnosed with SSUD, with ages ranging from 18 to 65 years (*Mean* = 31, *SD* = 3.7). The patients who were diagnosed with SSUD were mainly using alcohol or cannabis. All participants were receiving residential treatment, and they were abstinent for at least 21 days. To receive services in the rehabilitation centers, participants had to meet the criteria (present at least six or more symptoms, ensuring the severity) of SUD according to the diagnostic and statistical manual of mental disorders (DSM-V). They had no other psychiatric disorders. The presence of such other psychiatric diseases was evaluated through an in-depth psychiatric investigation conducted by a qualified psychiatrist. The demographic characteristics of both groups were matched as much as possible. Divisions of the sample are given in Table 1.

After obtaining approval from the ethics committee of the first author’s university, a researcher contacted site managers at rehab centers for permission to distribute the survey questionnaires to the participants. Eleven different rehabilitation centers were approached for data collection and debriefed about the study procedures by the researcher. Once participants were aware of the study’s procedures, they signed the informed consent form and completed the survey and demographic questionnaire. This study was not time-bound; however, it took 15–20 min to complete the questionnaires. Participants were not compensated to take part in this study. All data were collected between September and February 2021.

### 2.2. Inclusion and Exclusion Criteria

Adults whose ages were above 18, were using services for the treatment of SUD, and were abstinent for at least three weeks were selected to take part in this study. Patients with a neurological disorder, chronic disease, in an early stage of treatment, outpatients, and patients who were not willing to participate were excluded from the study.

#### 2.2.1. Measures/Instruments

One author physically visited the hospitals/rehab clinics and explained each item of the questionnaire to the participants in Urdu. This researcher is a Ph.D. scholar and lecturer at a university. He is also fluent in the English language and a native Urdu speaker.

#### 2.2.2. Demographic Form

We designed a demographics questionnaire with eight questions and included it in the survey. Age, education, occupation, family structure, gross monthly income, marital status, preferred drug, and the number of relapses were all covered in the questionnaires. The age of participants was asked as a quantitative variable. Education, occupation, family system, monthly income, and marital status were measured as categorical variables. We asked participants for their choice of drug. They reported different types of drugs, for example cocaine, opioids, cannabis, amphetamines, alcohol, heroin, tranquilizers, ecstasy, etc. We then classified these substances into four categories: stimulants (which increase the speed at which the brain and body exchange messages), depressants (which slow down these messages), painkillers (which relieve the symptoms of pain), and hallucinogens (which change the sense of reality).

#### 2.2.3. State Shame and Guilt Scale (SSGS)

The 10 items of two subscales from the State Shame and Guilt Scale (SSGS) developed by [45] were used to measure shame and guilt. The scale comprised 15 items and 3 subscales (shame, guilt, and pride). Each subscale consisted of five items. Example items were “I feel like I am a bad person.” and “I cannot stop thinking about something bad I have done.” The SSGS is based on a five-point Likert-type scale with response categories including 1 = *Not feeling this way at all* to 5 = *Feeling this way very strongly*. Participants rated their responses on the scales based on how they were feeling at that moment. The scores on the scale were interpreted as lower and higher scores. The alpha coefficient for the two subscales was stronger. In the polysubstance users, shame = 0.89 and guilt = 0.80. In the single-substance users, shame = 0.91 and guilt = 0.84.

#### 2.2.4. General Self-Efficacy Scale (GSE)

The General Self-Efficacy Scale (GSE) developed by [46] was used to measure the self-efficacy of both groups. The GSE consists of 10 items rated on a four-point Likert-type scale (1 = *not at all true* to 4 = *exactly true*). An example item was “I am confident that I could deal efficiently with unexpected events”. Minimum–maximum score ranges for the scale are 10 to 40. The higher scores on the scale indicate a high level of self-efficacy. The Cronbach’s alphas for the polysubstance users and the single-substance users were 0.87 and 0.90, respectively.

#### 2.2.5. Relapse Rate

Participants were questioned in an open-ended manner to measure their risk of relapse, for example: “How many times have you been in the hospital for addiction treatment?”. The range of relapse rate in our data was 1–5. For further statistical analysis, we categorize relapse rates into three categories, which are one time, two times, and three or more times. The mean relapse rate for people with PSUD were *M* = 2.83, *SD* = 0.85 and for people with SSUD were *M* = 1.9, *SD* = 0.78.

### 2.3. Statistical Analysis

First, descriptive statistics were applied to characterize the demographic features in more detail (Table 1). Using the SPSS 26 version, a one-way analysis of variance (ANOVA) was used to determine whether there were significant variations in the relapse rate among respondents with various demographic factors between those with PSUD and those with SSUD. Further, to determine the correlation between key variables, spearman correlation was performed.

Second, using mediation analysis [47], this study investigated the mediating function of guilt-proneness between shame-proneness and relapse rate. We also calculated the proportion of mediation, or how much of the overall effect of shame-proneness on relapse rate is mediated by guilt-proneness. The mediation proportion was calculated as = (ab)/c [48].

Third, the current study looked at how self-efficacy as a moderating variable influenced the direct impact of shame-proneness on relapse. Using the Hayes’ process macro (Model 5), a mediation moderation analysis was performed. The significance of all effects was also tested using the bootstrap approach to determine the standard error of parameter estimation [49]. From 5000 resampled data points, the bootstrap approach creates 95% bias-corrected confidence intervals for these effects. Confidence intervals that do not include zero denote significant influence.

## 3. Results

### 3.1. Descriptive Statistics and Correlation Analysis

The study was conducted on age-matched participants between people with polysubstance-use disorder (PSUD) and people with single-substance-use disorder (SSUD) with a mean (*M*) age of the people with PSUD of *M* = 32, *SD =* 5.5 and with SSUD of *M* = 31, *SD* = 3.7. Overall, people diagnosed with PSUD had a higher mean score of relapse rate than people with SSUD, with mean relapse rates for PSUD of *M* = 2.8, *SD* = 0.85 and mean relapse rate for SSUD of *M* = 1.9, *SD* = 0.78, respectively. Comparatively, the mean shame (*M* = 3.4, *SD* = 0.66) and mean guilt (*M* = 2.8, *SD* = 0.55) levels for people with SSUD were lower than those with PSUD (*M* = 4.9, *SD* = 0.34 and *M* = 4.8, *SD* = 0.44, respectively). People diagnosed with SSUD had greater self-efficacy scores (*M* = 3.6, *SD* = 0.93) than those with PSUD (*M* = 2.5, *SD* = 0.80). For people with PSUD, the majority of participants (30.3%) had a college degree, but for those with SSUD, the majority of participants were post-graduates (32.0%). Regarding employment, the majority of participants—both with PSUD (65.1%) and with SSUD (66.4%)—were unemployed. Most participants—both with PSUD and with SSUD—belong to joint families when family systems are considered (72.1% vs. 69.2 %, respectively). Some 70.1% of people with PSUD and 67.3% with SSUD reported that they had incomes of less than 30 thousand. Most with PSUD were married (55.7%), while most with SSUD were single (61.9%). With both PSUD and SSUD, the majority of participants reported painkillers as their drugs of choice (39.1% vs. 61.0%, respectively). These results are presented in Table 1. The findings of the correlation analysis indicated that relapse rate was significantly positively associated with shame-proneness and negatively associated with guilt-proneness and self-efficacy between both people with PSUD and SSUD. Shame-proneness is positively associated with guilt-proneness, and negatively correlated with self-efficacy between both groups. Guilt-proneness was positively associated with self-efficacy (Table 2).

### 3.2. Differences in Relapse Rate among Sociodemographic Factors

One-way analysis of variance (ANOVA) was carried out for the mean comparison by sociodemographic characteristics on the relapse rate between people with PSUD and SSUD participants. The results demonstrate significant mean differences in relapse rates across age groups. Younger participants reported high mean scores for relapse rate between both PSUD and SSUD participants. Comparing by education level, participants who have a low level of education reported high scores for relapse between both PSUD and SSUD. Furthermore, participants who belong to nuclear families reported high scores for relapse. Additionally, those who preferred painkillers as their drug of choice revealed high mean scores for relapse for both PSUD and SSUD participants (Table 3).

#### Mediation Moderation Model Test

The mediation moderation model was tested using Model 5 in the SPSS macro developed by [49]. Age, education, and the choice of the drug were considered as control variables in our analysis. Firstly, we looked at the direct impact of shame-proneness on relapse rate (Hypothesis 1). The results (Table 4) show shame-proneness substantially positively predicts relapse rate in people who are diagnosed with PSUD (*β* = 0.14, *t* = 11.76, *p* < 0.01), as well as those with SSUD (*β* = 0.05, *t* = 9.61, *p* < 0.01). Second, we considered whether shame-proneness might positively predict guilt-proneness, and whether guilt-proneness has a significant negative predictive effect on relapse rate (hypothesis 2). Results support the predictions by demonstrating a substantial positive effect of shame-proneness on guilt-proneness (*β* = 0.10, *t* = 12.23, *p* < 0.01), and guilt-proneness negatively predicts relapse (*β* = −3.05, *t* = −22.43, *p* < 0.01) in people with PSUD. These results were also significant in those who were diagnosed with SSUD: shame-proneness positively predict guilt-proneness (*β* = 0.09, *t* = 9.27, *p* < 0.05), and guilt-proneness negatively predicts the risk of relapse rate (*β* = −0.01, *t* = −19.58, *p* < 0.05). In the study results “*β*” is the standardized coefficient. These results demonstrate that guilt-proneness mediates the influence of shame-proneness on the relapse rate of individuals who use the drug.

When comparing these effects, we found a greater direct effect of shame-proneness on relapse rate and mediation effect of guilt-proneness in people with PSUD than in those with SSUD. Neither the 95% bootstrap confidence interval for the direct effect of shame-proneness on the relapse rate nor the mediating effect of guilt-proneness contained zero, showing that shame-proneness not only directly predicted relapse rate but also predicted relapse rate through the mediating effect of guilt-proneness.

**Figure 2:**
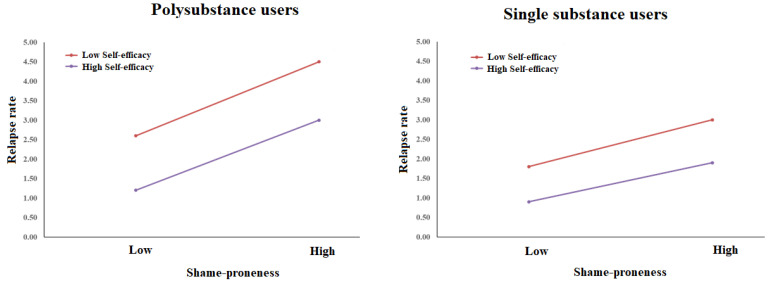
Moderating effect of self-efficacy on the relationship between shame and relapse rate between people with PSUD and SSUD.

In addition, we observed the interaction impact of shame-proneness and self-efficacy, which showed that when self-efficacy was higher vs. lower, the positive association between shame-proneness and relapse rate was weaker (hypothesis 3). Both those with PSUD and those with SSUD supported hypothesis 3 (*β* = −0.29, *t* = −5.99, *p* < 0.05 and *β* = −0.34, *t* = −13.49, *p* < 0.01, and respectively). A simple slopes analysis was used to investigate the moderating role of self-efficacy in the relationship between shame-proneness and relapse rate. As shown in Figure 2, in those with PSUD, shame-proneness was a positive predictor of relapse rate when self-efficacy was higher vs. lower (simple slope: b = −0.17, *t* = −10.23, *p* < 0.05), but not when self-efficacy was lower vs. higher (simple slope: b = 0.27, *t* = 09.13, *p* > 0.05), and similar results were found in those with SSUD when self-efficacy was higher vs. lower (simple slope: b = −0.24, *t* = −12.43, *p* < 0.01), but not when self-efficacy was lower vs. higher (simple slope: 0.11, *t* = 12.08, *p* > 0.05).

## 4. Discussion

Contrary to the popular belief that public shaming promotes positive behavioral change, scientific studies associate the emotions of shame and guilt with strengthened problem behavior. Emotions such as shame and guilt have been known to have an impact on SUD. However, the existing literature provides an extremely limited understanding of specific negative emotion that elicits patterns of substance use and potential relapse after treatment [50]. Further, comparative studies on people with PSUD and those diagnosed with SSUD are limited in the existing literature. So, our study discovered the positive association between shame and relapse rate via guilt-proneness, and also quantified how self-efficacy affects this relationship. Moreover, these effects were significantly stronger among people with PSUD than those with SSUD.

The findings from the current study reveal a significant positive association between shame-proneness and relapse rate among people with SUD; further, this association is stronger in people with PSUD as compared to people with SSUD. This may be because people with SUD take drugs to cope with negative self-conscious emotions, i.e., shame. The more they take drugs to get relief from their shame-based feelings, the more they experience shame. Using drugs is the only quick way to get short-term relief from these painful emotions [51]. This acting out then becomes the cause of negative consequences and creates even more uncomfortable feelings of shame. The existing literature specifying the relationship between shame-proneness and relapse rate in people with PSUD and SSUD is scarce in Pakistan. The results of this study thus strengthen the evidence of a positive correlation between shame-proneness and relapse rate. This is consistent with previous research that showed that shame-proneness puts people at a greater risk of relapse [23,28]. Further, shame-proneness has also been identified as a barrier to stopping the use of drugs such as stimulants [50]. The studies further indicate that shame-proneness does not support repairing a positive sense of self and therefore has little to no contribution to preventing relapses [52]. A previous study investigated the role of two complex human emotions, guilt and shame, in the field of substance abuse. The authors discovered that sedative drugs can induce internal pain, which is crucial to feelings of guilt and shame [53]. The results were in line with other research demonstrating that shame is a strong predictor of alcohol and other substance-use issues. Furthermore, it was evident that shame rather than guilt was a strong predictor of problematic addictive behavior [14,15,26]. Shame measures were more significantly linked to problematic behavior than guilt measures.

Another study on the correlation between shame and relapse in individuals with alcoholism problems found that discussing prior alcohol history was a powerful predictor of relapse propensity, severity, and impairments in health [26].

Further, when comparing people with PSUD and those with SSUD, one author proved that people with higher levels of PSUD were more likely to relapse and needed more residential and rehab treatment [1]. Moreover, people who used multiple drugs had more psychiatric problems including impulsivity, hyperactivity, and aggressivity [54]. According to the results of these earlier studies, shame propensity does not reduce but rather increases the risk of relapsing into a substance-use disorder. Cognitive theories of addiction explain that reliance on drugs as a mood adjuster can lead to the development of SUD (Wright et al., 1993). Activations of shame and guilt may be to blame for inner judging voices, which may require one to learn adaptive behaviors in order to silence them.

The results of the current study indicate that guilt-proneness mediates the relationship between shame-proneness and relapse rate among substance users. Further, these effects are stronger in people with PSUD as compared to people with SSUD. Because Pakistan is an Islamic country, it is illegal to use drugs. The norms of society and communities are still determined by the attitude and perspectives of community members and the larger society [55]. Further, people in recovery from SUD face more pressures than before. Long-term social stigma forces individuals to adopt a negative coping strategy, which increases the likelihood of negative emotions. Highly efficacious individuals try to transform negative emotions into adaptive behavior. Therefore, guilt also contributes to the reduction of relapse rate.

In such a context, guilt has played a stronger mediating role between shame-proneness and relapse. The results are consistent with past studies in which shame has been associated with avoidance in terms of behavior and guilt has been associated with eliciting a reparative response such as an apology [13]. Tangney et al. (2009) described guilt as a negative condemnation of a single act of behavior [32]. Meehan et al. (1996) associated guilt with functions to maintain attachment [14]. A study carried out on people recovering from alcoholism indicated that guilt-proneness was indirectly proportional to relapse [15]. Moreover, higher guilt-proneness was associated with lower chances of relapse [10]. The common consensus is that guilt highlights features of the individual’s actions, reflecting on a person’s perspective on how their behavior impacts others [28]. Dearing et al. (2005) pointed out that guilt-proneness provides the positive impetus for constructive change while promoting pro-social behavior such as apologizing and following corrective action [12].

Addiction is termed a family disease. On the one hand, stigmatization impacts people with SUD when they perceive poor emotional interactions among family members; and on the other hand, the indifferent and negative attitudes of family members can lead the person to assume that everyone rejects them. Individuals with SUD do not receive emotional support from family members, despite the fact that family members are the most significant and influential social support network [56]. This can cause a person to lose faith in their ability to succeed in recovery, which in turn makes it harder to cope with negative feelings and increases the risk of relapse.

The results of the current study indicate that self-efficacy moderates the effect of shame-proneness on relapse rate among people with PSUD and those with SSUD. Further, there are differences in the effect of shame-proneness on relapse rates at different levels of self-efficacy. When the level of self-efficacy of people with SUD is low, the predictive effect of shame-proneness on relapse rate is stronger; conversely, when the level of self-efficacy of people with SUD is high, the predictive effect of shame-proneness on relapse rate is weaker. Moreover, results indicate that people with SSUD reported a higher level of self-efficacy as compared to people with PSUD. This is because self-efficacy beliefs serve a special function in the study of addictive behaviors. Such beliefs affect both the early stages of SUD as well as the process of changing behavior that entails quitting such addictions and maintaining abstinence. In addition, self-efficacy plays a significant role in determining how well people respond to treatment for SUD. The findings of this study are consistent with earlier studies in showing that individuals with higher levels of self-efficacy view relapse as a temporary setback and make efforts to regain self-control, whereas individuals with lower levels of self-efficacy are more likely to experience a full relapse [57].

Self-efficacy was found to predict abstinence independently of drug urinalysis and treatment conditions during treatment for youths with substance-use disorders [58]. The moderating impact of self-efficacy on relapse was examined by Liu et al. (2020). The authors indicated that those with greater levels of self-efficacy reduced their risky behavior for relapse when given access to informational support, whereas those with lower levels of self-efficacy increased their risky behavior when given access to emotional support [59]. General self-efficacy predicted a significantly higher probability of no drug use and no heavy drinking after treatment. Moreover, self-efficacy was associated with fewer days of heavy drinking among people with SUD [60]. A lower level of self-efficacy belief in one’s ability to manage excessive drinking while experiencing unpleasant emotional states before treatment was associated with a greater level of alcohol dependence and an increase in the number of alcohol-related outcomes [61]. People with SUD who have a high level of self-efficacy are more adept at dealing with and mitigating the effects of negative emotions such as shame and guilt. They are also more likely to use positive reframing strategies to tackle challenges head-on, look for the positive side, and move on.

In Pakistan, there are far fewer treatment centers for substance use than are required to meet demand. Many of the hospitals that do exist offer unsatisfactory services that are not supported by evidence and are out of the price range of the majority of Pakistanis. Stigma, the recurring nature of the condition, and negative social attitudes toward addiction all contribute to keeping people away from treatment [62]. Due to many obstacles, only 33,000 Pakistanis obtained substance misuse treatment in 2013 (UNODC, 2013). Most people who obtain treatment for substance-use disorders nevertheless relapse thereafter. The relapse rate is approximately 90% in Pakistan [63]. The use of psychosocial therapies is often accepted in countries like Pakistan, where opioid substitution therapy or agonist maintenance therapy such as using methadone is illegal [64]. As a result, there may be long-term benefits to promoting personality-targeted therapy to aid in the recovery of persons who are affected by substance-use disorders. The findings of this research will thus help to build more successful and evidence-based rehabilitation programs in Pakistan for those who suffer from substance-use disorders.

### 4.1. Limitations and Future Studies

This study exclusively focused on male participants with substance-use disorder. Therefore, the results might not be applicable in general until further research is conducted. Furthermore, all the participants were receiving institutionalized care for rehabilitation. So, the results might vary among those who have not received institutionalized care. The relationship between emotions such as shame, guilt, and relapse is still poorly understood. Various studies conducted on shame and guilt have contrasting results. This will require a longitudinal study to shed more light on the causes and consequences of associations between these variables. Some of the findings of this study would benefit from further qualitative studies. The mediating effect of guilt-proneness on shame-proneness and predicting subsequent relapse would benefit from further support from qualitative data for understanding the dynamics of these variables. Moreover, further studies are needed to determine the causes behind people with PSUD being more prone to such relapses than people with SSUD.

### 4.2. Implications

The results of this study have both theoretical and practical implications. A significant implication is that the relationship between two measurable emotions, namely shame and guilt, provides researchers with a basis to predict a possible relapse and ways to prevent it. At a theoretical level, the findings enhance the understanding of the relationship between shame-proneness, guilt-proneness, and other underlying mechanisms such as their relationship with relapse and self-efficacy. The evidence on the interaction between shame-proneness and relapse rate has a direct implication on theories related to relapse among PSUD and SSUD. The finding on mediating the role of guilt-proneness between shame-proneness and relapse rate provides valuable insight into factors influencing relapse in people with PSUD and SSUD, which previously remained unexplored among this group. The results further strengthen the moderating role of self-efficacy between shame-proneness and relapse. This finding addresses the gap in previous studies where the relationship between shame and failure such as a relapse with more shame-eliciting behavior was not established.

At a practical level, it provides insights into aspects to focus on while supporting people with PSUD and SSUD in rehabilitation to prevent relapse. It is widely acknowledged that addiction treatment should be a tailored therapy based on the degree of shame-proneness and guilt-proneness of an individual. It emphasizes the need for assessing shame-proneness and guilt-proneness for each individual during substance-use-disorder treatment. Relating to this practice, the results of this study will inform rehabilitation practitioners about supporting individuals with PSUD and SSUD based on empirical evidence on psychological traits such as self-efficacy. Individuals who have lower self-efficacy might need more support to address emotions such as shame-proneness to prevent their risk of relapse. Thus, the results of this study will support the design of more effective and evidence-based rehabilitation programs for people with substance-use disorder.

## 5. Conclusions

The results of this study demonstrate the complex relationship between emotions and their impact on substance-use disorder and the possibility of subsequent relapse. This study drew the following conclusions: (1) In general, those with PSUD report higher levels of shame-proneness and guilt-proneness, lower levels of self-efficacy, and are more likely to relapse than people with SSUD. (2) Shame-proneness positively predicted relapse rate in people with PSUD. (3) Guilt-proneness mediated the relationship between shame-proneness and relapse rate. (4) The direct effect of shame-proneness on relapse rate was moderated by self-efficacy. Compared with substance users who had low self-efficacy, substance users with high self-efficacy had less vulnerability to relapse. Although the mediation and moderation effects were found in both study groups, these effects were significantly stronger among people diagnosed with PSUD than those with SSUD. To be more specific, those diagnosed with PSUD reported a higher overall score on shame-proneness, guilt-proneness, and relapse rate than those with SSUD. The findings of this study suggest that people with PSUD are more likely to experience shame, guilt, and relapse than people with SSUD. Drug rehab facilities should implement a variety of strategies to raise drug users’ levels of self-efficacy, which will help to reduce their risk of relapse.

## Figures and Tables

**Table 1:** Sociodemographic characteristics between the people with PSUD and SSUD.

Variables	People with PSUD (*n* = 402)		People with SSUD (*n* = 410)		*p*-Value
	*n*	*%*	*n*	*%*	
Age (mean± SD)	32 ± 5.5		31 ± 3.7		0.471
Relapse rate	2.8 ± 0.85		1.9 ± 0.78		<0.01
Self-efficacy (mean± SD)	2.5 ± 0.80		3.6 ± 0.93		<0.001
Shame level (mean± SD)	4.9 ± 0.34		3.4 ± 0.66		<0.001
Guilt level (mean± SD)	4.8 ± 0.44		2.8 ± 0.55		<0.001
**Education**					
High school	98	24.3	61	15.1	<0.01
College level	122	30.3	129	23.1	
Graduate	105	26.1	127	31.5	
Post-graduate	77	19.1	93	32.0	
**Occupation**					
Students	20	4.9	24	5.8	<0.01
Employed	120	29.8	114	27.8	
Unemployed	262	65.1	272	66.4	
**Family system**					
Joint	290	72.1	284	69.2	<0.01
Nuclear	112	27.8	126	30.8	
**Monthly income**					
Less than 30 K	282	70.1	276	67.3	<0.001
30–60 K	83	20.6	71	17.3	
61–90 K	22	5.5	38	9.3	
Above 90 K	15	3.8	25	6.1	
**Marital status**					
Married	224	55.7	156	38.1	<0.001
Single	178	44.3	254	61.9	
**Drug of choice**					
Stimulants	69	17.2	48	11.7	<0.01
Depressants	93	23.1	55	13.4	
Painkillers	157	39.1	250	61.0	
Hallucinogens	83	20.6	57	13.9	

Note. A comparison of qualitative variables’ proportion between people with PSUD and SSUD was performed using a chi-square test, and quantitative variables’ mean between groups was compared using a *t*-test.

**Table 2:** Correlation of sociodemographic characteristics with self-evaluative emotions, relapse rate, and self-efficacy among people with PSUD and SSUD.

Variables	1	2	3	4	5	6	7	8	9	10	11
1. Relapse rate	-	0.51 **	−0.36 **	−0.34 *	0.15 *	0.08 *	0.05	0.24	0.69 *	0.91 *	0.12
2. Shame-proneness	0.79 **	-	0.48 **	−0.41 **	0.14	0.31 *	0.13 *	0.19 *	0.05	0.13	0.35 *
3. Guilt-proneness	−0.47 **	0.59 **	-	0.32 *	0.10	0.37 *	0.41 *	0.23 *	0.07	−0.03	0.41
4. Self-efficacy	−0.63 *	−0.45 **	0.65 *	-	0.08 *	0.19 *	0.07	0.03	0.11	0.81	0.62
5. Age	0.18 *	0.07	0.09	0.12	-	0.17	0.15	−0.04	0.13	0.31	0.10
6. Education	0.28 *	0.19	0.26 *	−0.20	0.15	-	0.25	−0.03	0.26	0.40	0.36
7. Occupation	0.14	0.56 *	0.49 *	0.05	0.01	−0.12	-	0.11 *	−0.09	−0.05	0.61
8. Family system	0.17 *	0.45 *	0.50 *	−0.03	−0.09	0.03	0.29	-	−0.05	0.10	0.02
9. Monthly income	0.98 *	−0.48 *	−0.36 *	−0.31	0.19	−0.08	0.04	−0.17	-	0.51	0.28
10. Marital status	0.93	0.03	0.06	0.21	−0.26	0.02	−0.21	−0.31	0.02	-	0.13 *
11. Drug of choice	0.90 **	0.29	0.38 *	0.45	0.72 *	0.42 *	0.26	−0.06	0.05	0.82	-

Note. The results for the sample of people with PSUD (*n* = 402) are below the diagonal. The results for the sample of people with SSUD (*n* = 410) are above the diagonal. * *p* < 0.05, ** *p* < 0.01.

**Table 3:** Mean comparison of relapse rate between people with PSUD and SSUD by sociodemographic factors.

		People with PSUD			People with SSUD		
		Mean ± SD	*F*	*p*	Mean ± SD	*F*	*p*
Age	Below 20 (yrs.)	1.66 ± 0.81	7.12	0.04	1.70 ± 0.79	11.73	0.03
	20–40 (yrs.)	2.90 ± 0.89			2.62 ± 0.86		
	41–60 (yrs.)	2.03 ± 0.81			2.03 ± 0.81		
	Above 60 (yrs.)	2.33 ± 0.81			2.23 ± 0.81		
Education level	High school	2.82 ± 0.88	10.40	0.03	2.71 ± 0.84	9.93	0.007
	College	2.12 ± 0.85			2.12 ± 0.85		
	Graduate	1.95 ± 0.82			1.95 ± 0.81		
	Post-graduate	2.04 ± 0.85			2.02 ± 0.85		
Occupation	Students	2.03 ± 0.87	0.49	0.92	2.04 ± 0.83	0.14	0.73
	Employed	2.01 ± 0.85			2.00 ± 0.85		
	Unemployed	1.99 ± 0.80			1.99 ± 0.80		
Family system	Joint	1.99 ± 0.76	8.06	0.04	1.48 ± 0.75	6.36	0.04
	Nuclear	2.81 ± 0.87			2.41 ± 0.86		
Income level	Below 30 K	1.99 ± 0.83	0.01	0.08	2.01 ± 0.82	2.07	0.85
	30–60 K	2.04 ± 0.81			2.04 ± 0.80		
	61–90 K	1.98 ± 0.91			1.89 ± 0.92		
	Above 90 K	2.00 ± 0.84			2.00 ± 0.84		
Marital status	Married	2.01 ± 0.82	1.70	0.12	2.00 ± 0.82	2.80	0.91
	Single	2.00 ± 0.84			2.01 ± 0.84		
Drug of choice	Stimulants	2.01 ± 0.84	17.32	0.02	1.83 ± 0.75	14.19	0.002
	Depressants	1.90 ± 0.84			1.93 ± 0.81		
	Painkillers	2.97 ± 0.89			2.65 ± 0.86		
	Hallucinogens	1.93 ± 0.77			2.02 ± 0.87		

**Table 4:** Mediation and moderation results of the variables between people with PSUD and SSUD.

		People with PSUD (*n* = 402)						People with SSUD (*n* = 410)					
Regression Equation		Overall Model Fit			Regression Coefficient Significance			Overall Model Fit			Regression Coefficient Significance		
DVs	IVs	R	R^2^	*F*	*ꞵ*	95%CI	*t*	R	R^2^	*F*	*ꞵ*	95%CI	*t*
Guilt-prone	Age	0.58	0.43	34.65	0.04	0.01,0.08	7.23	0.48	0.35	23.7	0.07	0.04, 0.09	11.21
	Education				−0.37	0.10,0.41	−5.42				0.41	0.14, 0.31	7.52
	Drug of choice				1.23	0.83,1.64	5.97				−0.3	−0.67, 0.76	−1.35
	Shame-prone				0.10	0.23, 0.54	12.23				0.09	0.56, 0.74	9.27
	Relapse rate				0.03	1.21, 2.33	16.54				0.01	2.12, 3.46	12.73
Relapse rate	Age	0.69	0.57	28.45	0.36	0.21, −0.78	0.75	0.52	0.61	21.5	0.23	−0.17, 1.34	−0.15
	Education				0.33	0.04, 0.43	9.65				0.16	1.43, 2.31	6.85
	Drug of choice				0.98	0.36, 1.76	16.53				0.47	1.33, 1.61	4.89
	Guilt-prone				−3.05	−5.91, −1.66	−22.43				−0.01	−0.74, −1.45	−19.58
	Shame-prone				0.14	0.01, 1.65	11.76				0.05	1.17, 1.57	9.61
	Self-efficacy				0.06	0.01, 0.77	7.82				1.08	1.25, 1.62	5.92
	Shame-prone × Self-efficacy				−0.29	−1.43, −1.97	−13.49				−0.34	−0.01, −0.09	−5.99

## Data Availability

The datasets processed and analyzed during the current study are available from the corresponding/first author on reasonable request.

## Abbreviations

SUD: substance-use disorder; PSUD: polysubstance-use disorder; SSUD: single-substance-use disorder
